# Inhibitory effect of *Zataria multiflora* Boiss. essential oil, alone and in combination with monolaurin, on *Listeria monocytogenes*

**Published:** 2016-03-15

**Authors:** Mojtaba Raeisi, Hossein Tajik, Seyed Mehdi Razavi Rohani, Bektas Tepe, Hossein Kiani, Rahem Khoshbakht, Hesamaddin Shirzad Aski, Hamed Tadrisi

**Affiliations:** 1 *Department of Public Health, Faculty of Health, Golestan University of Medical Sciences, Gorgan, Iran; *; 2 *Cereal Health Research Center, Golestan University of Medical Sciences, Gorgan, Iran; *; 3 *Department of Food Hygiene and Quality Control, Faculty of Veterinary Medicine, Urmia University, Urmia, Iran; *; 4 *Department of Molecular Biology and Genetics, Faculty of Science and Literature, Kilis 7 Aralik University, Kilis, Turkey;*; 5 *Bioprocessing and Biodetection Laboratory, Department of Food Science, Faculty of Agricultural Engineering and Technology, University of Tehran,Karaj, Iran; *; 6 *Department of Food Hygiene and Public Health, Faculty of Veterinary Medicine, Amol University of Special Modern Technologies, Amol, Iran; *; 7 *Department of Pathobiology, School of Veterinary Medicine, Shiraz University, Shiraz, Iran; *; 8 *Graduated of Veterinary Medicine, Faculty of Veterinary Medicine, Urmia University, Urmia, Iran.*

**Keywords:** Essential oil, *Listeria monocytogenes*, Minimum inhibitory concentration, Monolaurin, *Zataria multiflora* Boiss

## Abstract

*Listeria monocytogenes* is one of the major causes of infections in developing countries. In this study, chemical composition and anti-listerial effect of the essential oil of *Zataria multiflora* Boiss. alone and in combination with monolaurin were evaluated at different pH values (5, 6, and 7) and temperatures (5 ˚C and 30 ˚C). Chemical composition of *Zataria multiflora* Boiss. essential oil was evaluated by gas chromatography-mass spectrometry (GC-MS) analysis. Minimum inhibitory concentration (MIC) of the essential oil and monolaurin were determined using microbroth dilution method and the interactions of essential oil and monolaurin were determined by the evaluation of fractional inhibitory concentrations (FIC) index. Carvacrol (63.20%) and thymol (15.10%) were found as the main components of the essential oil. The MIC values of the oil and monolaurin at pH 7 and 30 ˚C were measured as 312.50 µg mL^-1^ and 125.00 µg mL^-1^, respectively. Combination of monolaurin and *Z. multiflora* essential oil were found to act synergistically (FIC index < 0.5) against *L. monocytogenes* under different pH and temperature conditions. Decrease in the pH and temperature values have increased the anti-listerial activity of monolaurin and the essential oil. The lowest MIC value of monolaurin and essential oil was observed at pH 5 and 5 ˚C. According to our results, the oil alone or in combination with monolaurin at low pH and temperature conditions showed a promising inhibitory effect on *L. monocytogenes*.

## Introduction

*Listeria monocytogenes* is one of the most foodborne pathogens that has been found in different environments including soil, water and food (especially in meat and dairy products).^1^ This gram-positive bacterium seems to be a major concern for consumers due to severe diseases caused by the bacterium such as abortion, meningitis and perinatal septicemia.^2^ One of the most important characteristics of this bacterium is its ability to grow at different conditions such as refrigeration temperatures, anaerobic conditions and conditions with low levels of oxygen.^2^^,^^3^ Safe chemical antimicrobials have been widely used for the preservation of food products. Monolaurin is one of the generally recognized as safe (GRAS) fatty acid derivative that shows strong antimicrobial activity among the widely varied fatty acid derivatives, possessing several useful characteristics such as emulsification properties and antimicrobial effect on gram-positive bacteria, yeasts and molds.^4^ However, it has been reported that gram-negative bacteria could not be affected by this anti-microbial agent. To enhance the effect of monolaurin on gram-negative bacteria, combination of this antimicrobial with chelating agents such as EDTA, food grade acids such as acetic acid or heat treatment should be used.^5^

In recent years, besides the use of chemicals, food preservation by natural products and preservatives has been considered as a new and safe approach for inhibiting the growth of food borne pathogens and spoilage bacteria.^6^ Spices and essential oils are considered as natural components for food industries.^7^ These materials usually exhibit different characteristics such as antimicrobial and flavoring effects.^7^ *Zataria multiflora *Boiss. (named as Avishan-e-Shirazi in Farsi) is belonging to Laminaceae family that grows in Iran, Pakistan and Afghanistan and this plant is widely used as a flavoring agent in several foods.^6^ Thymol and carvacrol, the main components of the essential oil of *Z. multiflora*, has been reported to have antibacterial, antioxidant, antiseptic and antifungal properties.^8^^-^^10^

The antibacterial effect of the essential oil of *Z. multiflora* and monolaurin has previously been reported against some bacteria. However, based on the knowledge of the authors, no report is available on their combination effect against gram-positive or gram-negative bacteria. The objective of this study was to evaluate the effect of monolaurin and *Z. multiflora *oil both alone and in combination against *L. monocytogenes *at different pH values and storage temperatures.

## Materials and Methods

**Antimicrobials** **and** **chemicals.** Monolaurin (Med-Chem Laboratories Inc., Galena, USA) stock solution was prepared by dissolving it in absolute ethanol (Sigma-Aldrich, Munich, Germany) to yield 1000 µg mL^-1^. *Zataria multiflora *was purchased from a local grocery store and was authenticated at the Faculty of Agriculture, Urmia University, Urmia, Iran. The essential oil (EO) was obtained from the aerial parts by hydrodistillation for 3 hr, using a clevenger-type apparatus. The EO was dehydrated with anhydrous sodium sulfate and filtered through a 0.22 μm filter (Millipore™, Bedford, USA) and stored at 4 ˚C for further analysis. Stock solution of the oil (10000 µg mL^-1^) was prepared by dissolving 0.20 g of the oil in 2.00 mL of brain heart infusion broth (BHI; Oxoid Ltd., Basingstoke, UK) containing 10% DMSO (Sigma-Aldrich). Culture media used in this study was BHI broth (Oxoid Ltd.), which adjusted to pH 5, 6 and 7 using citric acid (Merck, Darmstadt, Germany) and NaOH (Merck). 

**Analysis of the essential oil.** The analysis of *Z. multiflora* essential oil was performed using an Agilent 6890N chromatographer (Agilent Technologies Inc., Santa Clara, USA) that was equipped with an HP-5MS capillary column (30 × 0.25 mm ID × 0.25 mm film thickness; Agilent Technologies Inc.). Carrier gas was helium with a flow rate of 1 mL min^-1^. The column temperature was initially 50 ˚C, and then gradually increased to 120 ˚C at a 2 ˚C min^-1^ rate, held for 3 min, and finally increased to 300 ˚C. The procedure was operated at 70 eV. The compounds were identified by comparing their retention indices with those of authentic samples and mass spectral data available in the library (Wiley registry, 10^th^ ed, National institute of standard and technology, 2014, mass spectral library).

**Test microorganisms.** The strain used in this study was *L. monocytogenes* ATCC 19118. Lyophilized cultures of the organisms were obtained from the culture collection of the Department of Microbiology, Faculty of Veterinary Medicine, Urmia University, Urmia, Iran.

**Preparation of serial dilutions of** ***Z. multiflora*** **essential oil and monolaurin.** Serial dilutions of essential oil were prepared in BHI broth from oil stock solution (10000 µg mL^-1^) to obtain different concentrations ranged from 9.70 to 5000 µg mL^-1^. A similar procedure but in different concentrations was used to prepare the serial dilutions of monolaurin including (500.00, 250.00, 125.00, 62.50, 31.25, 15.62, 7.81, 3.90, 1.95, 0.97, 0.48 and 0.24 µg mL^-1^).

**Minimum inhibitory concentration (MIC) of the monolaurin and essential oil alone.** Minimum inhibitory concentration of monolaurin was determined using micro-broth dilution method. For this purpose, 96 well micro-plates were used. A volume of 160 µL of BHI broth with particular pH adjusted with HCL (5, 6 and 7), 20 µL of different concentrations of monolaurin and 20.00 µL of BHI broth containing 10^5^ CFU per mL of *L. monocytogenes* were added into each well. The last well containing 180.00 µL BHI broth and 20.00 µL of inoculum without monolaurin was designed as the positive control. For negative control, un-inoculated BHI broth was used in order to determine sterility. Contents of each well were mixed using a plate shaker at 300 rpm for 30 sec and afterwards, microplates were incubated at two different temperatures (5 ˚C and 30 ˚C) for 48 hr. The microbial growth was assessed by using ELx 800 universal micro-plate reader (Biotek Instrument Inc., Winooski, USA) by reading the absorbance of each well at 600 nm. The MIC was defined as the lowest concentration of the anti-microbial that prevented the growth of *L. monocytogenes *completely. To determine MIC of *Z. multiflora* essential oil at different pH values and temperatures, the procedure presented above was used, but the concentrations of the oil were adjusted at different levels including: 5000, 2500, 1250, 625.00, 312.50,156.20, 78.10, 39.05, 19.50, 9.70, 4.80, and 2.40 µg mL^-1^).

**Assessment of monolaurin and essential oil interactions.** Fractional inhibitory concentrations (FIC) index was used to determine the antimicrobial effect of monolaurin and essential oil combination. FIC index was defined as follows:

FICI = FICAMICA in combinationMICA alone+FICBMICBin combinationMICB alone

The FICI was defined as described previously by Fei *et al*.:^11^ A synergistic effect when FICI ≤ 0.5, an additive effect when 0.5 < FICI < 1, an antagonism effect when FICI > 4, without effect when 1 < FICI < 4. 

## Results

**Chemical composition of** ***Z. multiflora*** **Boiss. essential oil.** Air dried parts of *Z. multiflora* Boiss. yielded 1.50% of essential oil. Essential oil composition of *Z. multiflora* is presented in Table 1. According to gas chromatography–mass spectrometry (GC-MS) analysis, 13 components were identified representing 96.80% of the total oil. The main components were phenolic mono-terpene carvacrol (63.20%), followed by thymol (15.10%) and -terpinene (2.70%).

**Table 1:** Composition of Zataria multiflora essential oil

**Groups**	**RI** *	**Percentage**
-Pinene	937	2.20
Linalool	1086	1.20
Carvacrol	1288	63.20
-terpinene	1055	2.70
Eucaliptol	1024	0.40
Globulol	1582	1.80
-Terpineol	1027	0.70
Mycerene	984	0.50
Carvacerol methyl ether	1228	2.30
*trans*-Caryophyllene	1428	0.50
Aromadendrene	1450	1.80
Thujene	928	0.10
Thymol	1270	15.10
Monoterpene hydrocarbons	-	2.20
Oxygenated monoterpenes	-	2.10
**Total**		96.80

* Retention index

**Antimicrobial effect of essential oil and monolaurin alone in different conditions.** Antibacterial effect of the essential oil and monolaurin are presented in Table 2. The MIC values of the oil and monolaurin at pH 7 and temperature of 30 ˚C against *L. monocytogenes* were 312.50 µg mL^-1^ and 125.00 µg mL^-1^, respectively. As it is shown in Table 2, by lowering the pH level to 5, the MIC values of the tested antimicrobials were decreased. The detected MIC of the essential oil and monolaurin at this pH value were 78.10 µg mL^-1^ and 62.50 µg mL^-1^, respectively. Reduction in incubation temperature led to a decrease in MIC values of the essential oil and monolaurin. For instance MIC of the oil and monolaurin at 5 ˚C and pH = 7 were estimated to be 78.10 µg mL^-1^ and 62.50 µg mL^-1^, respectively. According to the results, reduction in the pH and incubation temperature led to an increase in the antilisterial effect of the antimicrobials (Table 2).

**Table 2:** Minimum inhibitory concentration of monolaurin and *Zataria multiflora* essential oil alone against *Listeria monocytogenes* at different pH values and temperatures

**Samples**	**pH and temperature variations**					
	**pH 7.0** **30 ** **˚C**	**pH 6.0** **30 ** **˚C**	**pH 5.0** **30 ** **˚C**	**pH 7.0** **5 ** **˚C**	**pH 6.0** **5 ** **˚C**	**pH 5.0** **5 ** **˚C**
**Essential oil **	312.50	156.20	78.10	78.10	156.20	78.10
**Monolaurin**	125.00	125.00	62.50	62.50	31.25	31.25

**Evaluation of antimicrobial interactions.** The results obtained in this study confirmed that satisfactory growth inhibition against *L. monocytogenes* could be achieved when a combination of essential oil and monolaurin were employed (Table 3). As presented in Table 3, the FIC index of the essential oil and monolaurin combination at 30 ˚C for both pH values of 5 and 7 was 0.09. The FIC indices of essential oil and monolaurin combination, at pH 5, 6 and 7 at 5 ˚C were 0.06, 0.12 and 0.04, respectively. Results of this section revealed that the interaction of the essential oil and monolaurin was a strong synergistic interaction in all test conditions, as the values of FIC were low. The FIC values of essential oil and monolaurin combination, at pH 5, 6 and 7 at similar 5 ˚C were 0.06, 0.12 and 0.04. 

**Table 3:** Fractional inhibitory concentration (FIC) index for the combination of *Zataria multiflora* essential oil and monolaurin against *Listeria monocytogenes* at different pH and temperatures

**Conditions**	**FIC** 1	**FIC** 2	**∑FIC** 3
**pH 7.0 + 30** **˚C**	0.06	0.03	0.09
**pH 7.0 + 5 ˚C**	0.03	0.01	0.04
**pH 6.0 + 30 ˚C**	0.03	0.06	0.09
**pH 6.0 + 5 ˚C**	0.06	0.06	0.12
**pH 5.0 + 30 ˚C**	0.06	0.03	0.09
**pH 5.0 + 5 ˚C**	0.03	0.03	0.06

The result of all different conditions were synergistic effect.

1 FIC for *Z. multiflora* essential oil;

2 FIC for monolaurin;

3 FIC for the combination of *Z. multiflora* essential oil and monolaurin.

## Discussion

*Zataria multiflora*, has been the subject of numerous studies through the food industry due to its remarkable antimicrobial activity. A number of these studies are carried out *in vitro*. Shariffar *et al*. and Saei-Dehkordi *et al*. reported *Z. multiflora* essential oil as a potent antimicrobial agent which inhibited growth of food borne pathogens.^12^^,^^13^ Also, a large number of these studies are intended to extend the shelf-life of foods by preventing microbial contaminations.^14^^,^^15^ Additionally, studies for determining the antimicrobial activity of *Z. multiflora* essential oil in combination with other components are also provided. Preservative effects of *Z. multiflora* essential oil (ZEO) at 0.02%, 0.05% and 0.10%, sodium acetate (SA) at 2.00%, and their combination on the quality changes of vacuum-packaged trout burgers during 21-days refrigerated storage (4 ± 1 ˚C) were investigated by Ehsani *et al*.^16^ According to this report, combined application of SA and ZEO extended the shelf life of fish burgers during cold storage to 21 days. In another study, the capabilities of *Z. multiﬂora* essential oil (0.03%, 0.06%, w/w), nisin (9.00, 18.00 mg kg^-1^), potassium sorbate (500 and 1000 mg kg^-1^) and low density polyethylene (LDPE) package containing 0.40% and 1.00% (w/w) nano-ZnO on shelf-life of caviar were investigated by Heshmati *et al*.^17^ According to this study, 0.06% (w/w) *Z. multiflora* essential oil showed the most significant effect on the shelf-life of caviar samples (*p *< 0.05).

Various studies have been conducted on the chemical composition and antimicrobial effect of essential oil of plants belonging to Laminaceae family, especially *Z. multiflora* Boiss. essential oil and in most of them, carvacrol and thymol are reported as the major compounds of the essential oil of *Z. multiflora.*^12^^,^^13^^,^^18^ Moosavy *et al*. reported carvacrol (71.20%), -terpinene (7.34%) and -pinene (4.26%) as the main compounds of the oil.^8^ According to another study, thymol (38.70%), carvacrol (15.30%) and *p*-cymene (10.20%) were determined as the major components.^19^ The variability and diversity of the reports regarding to chemical composition of *Z. multiflora* Boiss. essential oil can be attributed to different geographical conditions, climate and seasonal variations and the stage of the plant growth.^20^ The antimicrobial activity of the *Z. multiflora* essential oil could be attributed to the presence of carvacrol and thymol. Carvacrol, a phenolic mono-terpene, has been reported as one of the most efficient plant antimicrobial agents. The mechanism of action of carvacrol is attributed to destabilization of the cytoplasmic membrane by this compound and its act as a proton exchanger reducing the pH gradient across the membrane.^21^ In addition, thymol, another major component of *Z. multiflora* essential oil can cross the cellular membrane, interact with membrane enzymes and proteins and affect the cellular activity. These antimicrobial effects has also been reported for carvacrol and other phenolic compounds.^22^

Antibacterial effect of the essential oil and monolaurin was evaluated by MIC determination. As mentioned in results section, in general, antibacterial effect of these components increased when the pH value and incubation temperature were reduced. The lowest MIC value was observed at pH 5 and 5 ˚C. The results obtained in this study showed that *Z. multiflora* essential oil exhibited strong antibacterial activity against *L. monocytogenes*. Anti-bacterial activity exhibited by the oil is possibly due to the presence of high amounts of thymol and carvacrol. According to the results obtained here, monolaurin also showed significant anti-listerial activity. MIC value of monolaurin was detected as 125.00 µg mL^-1^ against *L. monocytogenes* at pH 7 and 30 ˚C. Monolaurin and other monoester fatty acids are the lipophilic substances and the inhibitory effect of monolaurin is probably due to its interference with cytoplasmic membrane of micro-organisms.^23^ By this way, damage in ion permeability control, deterioration of protein components such as enzymes and excretion of the intracellular constituents occurs.^24^^,^^25^ Results presented in this study are in agreement with previous studies.^26^^-^^28^ Oh and Marshal reported that monolaurin had the highest antibacterial activity among all fatty acids and their esters tested by the authors.^4^ The combined usage of the oil and monolaurin was evaluated in this study in different pH value and temperatures. As it mentioned above, combination of monolaurin and *Z. multiflora* oil were found to act synergistically (FIC index < 0.50) against *L. monocytogenes* under different pH and temperature conditions.

In conclusion, the present study indicated that *Z. multiflora* essential oil and monolaurin showed remarkable antibacterial activity against *L. monocytogenes* and the combination of these components was revealed to be a more potent inhibitor against this bacterium. Synergistic antimicrobial effects were detected for these two agents. So, monolaurin and *Z. multiflora* essential oil could be considered as potential strong antimicrobials for the growth inhibition of *L. monocytogenes* in food products.
